# New Patterns of Information and Communication Technologies Usage at Work and Their Relationships with Visual Discomfort and Musculoskeletal Diseases: Results of a Cross-Sectional Study of Spanish Organizations

**DOI:** 10.3390/ijerph16173166

**Published:** 2019-08-30

**Authors:** María Soria-Oliver, Jorge S. López, Fermín Torrano, Guillermo García-González, Ángel Lara

**Affiliations:** 1 Facultad de Ciencias de la Salud, UNIR-Universidad Internacional de la Rioja, Av. de la Paz, 137, 26006 Logroño, Spain; 2 Departamento de Ciencias de la Salud, Facultad de Ciencias de la Salud, Universidad Pública de Navarra, Campus de Arrosadía s/n, 31006 Pamplona, Spain; 3 Escuela Superior de Ingeniería y Tecnología, UNIR-Universidad Internacional de la Rioja, Av. de la Paz, 137, 26006 Logroño, Spain; 4 Facultad de Derecho, UNIR-Universidad Internacional de la Rioja, Av. de la Paz, 137, 26006 Logroño, Spain; 5 Instituto Nacional de Seguridad y Salud en el Trabajo, CNNT. C/Torrelaguna, 73, 28027 Madrid, Spain

**Keywords:** information and communication technologies, exposure combinations, ergonomics, work organization, musculoskeletal diseases, visual discomfort, cross-sectional study

## Abstract

This cross-sectional study analyses the usage patterns of the new communication and information technologies (ICTs) and their relationship with visual discomfort and musculoskeletal diseases in an intentional sample of 1259 workers of Spanish organizations. The usage pattern with the greatest incidence of visual and muscular-skeletal disorders, especially in the wrist and neck, combines the use of laptops and desktops during long working hours. However, the group of workers primarily using mobile devices and working mostly at mobile posts does not appear to be particularly vulnerable to the musculoskeletal diseases and visual fatigue. The ratio of taking a short pause per hour and the implantation of certain technical and preventive measures is related to lower incidence of disorders in the workers as a whole. Current usage of ICTs is very complex and should be addressed using empirical analysis of the different forms of usage and their impacts on health.

## 1. Introduction 

The development of new information and communication technologies (ICTs) is having a profound impact on work environments. As shown in recent reports, laptops, tablets, and smartphones have gradually been incorporated as working tools in different professional sectors [1,2]. This implementation runs parallel to a progressive modification in the ways of organizing work, which affects different aspects of working and personal life [3,4].

The study of the usage patterns of mobile devices in the work setting and the analysis of their effects is still an emerging field [5,6]. However, various authors agree that the possibilities offered by the new ICTs are a double-edged sword [4,7]. In this sense, despite their potential advantages, extending work to different environments linked to the development of mobile devices involves diverse risks. With regard to the physical consequences, some studies have already shown the relationship between the use of mobile devices and certain musculoskeletal disorders (MSDs). Thus, excessive neck flexion posture and thumb joint strain typical of mobile device usage have been reported as contributing factors to MSDs [8,9,10]. Ciccarelli et al. [11] found that workers’ combined ICT tasks possibly presented the greatest risk for developing musculoskeletal complaints. Woo et al. [6] noted a high prevalence of MSDs among university students who used computers and other electronic devices daily over long periods of time. They also observed associations between demographic and ergonomic factors and MSDs prevalence. However, Palmer et al. [12] found no statistical association between the level of exposure to ICTs and discomfort reported in adolescents. So et al. [5] also found only moderate relationships between cumulative mobile device and MSDs in the general adult population. The recent review of Xie et al. [13] on the use of mobile devices despite showing that complaints in the neck, shoulders, thumbs, and fingers, compared with other body regions, are more commonly reported among users of handheld mobile devices, also reveals a very broad range of prevalences that is compatible with similar wide ranges of period or lifetime prevalence for neck and upper limb complaints in the general population. However, to our knowledge, there are no studies that analyze the use of different ICT devices globally and their physical consequences in the work setting.

Thus, we should advance in our knowledge of the new usage patterns of ICTs in the work setting, analyzing both the specific use of different devices and their mobility dimension and observing their effects through indicators of visual and MSDs. Taking these considerations into account, this work aims to provide a more precise comprehension of the current use of ICTs in the work setting, covering the following objectives: (1) empirically exploring which patterns of combined use of ICTs occur in the current work setting; (2) analyzing the relationships between the different ICT usage patterns in the work setting and the incidence of musculoskeletal and visual disorders; (3) analyzing the relationship between the incidence of the aforementioned disorders and the technical and formative preventive measures implemented in the work setting. 

## 2. Conceptual Framework and Hypothesis

Workers’ ICT usage pattern is conceptualized as a combination of the type of terminals used, the duration of use of each one, and the mobile or fixed nature of the job. These patterns will be defined empirically through grouping exploratory techniques, as specified below. 

**Figure 1:**
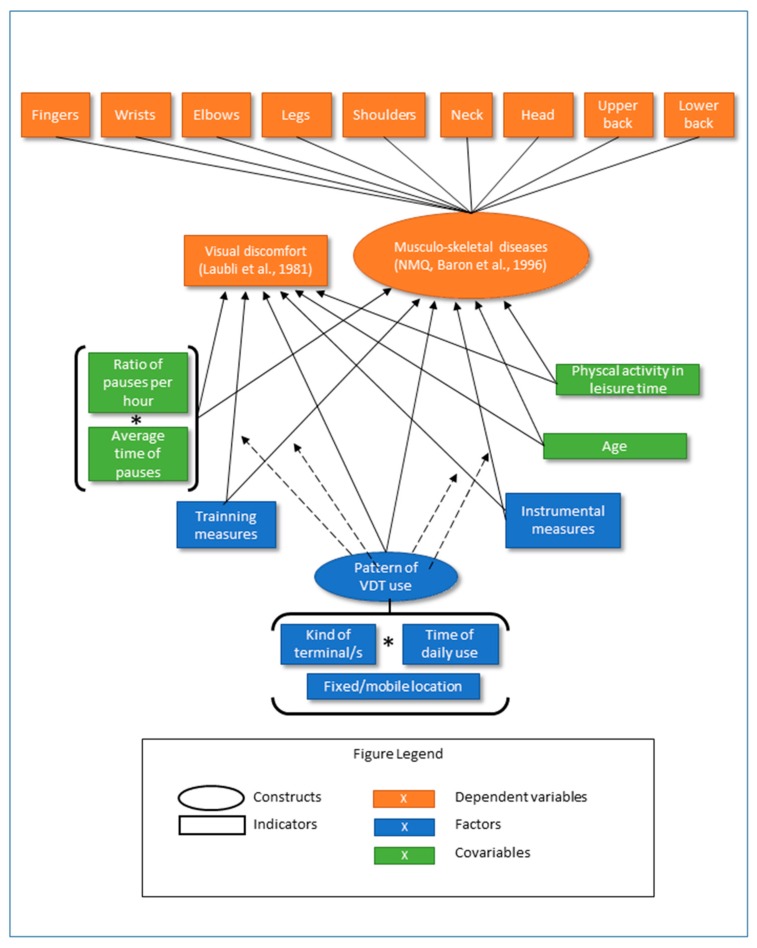
Conceptual framework and structure of relations between indicators.

The conceptual framework and the relationship hypothesis proposed for the study are shown in Figure 1. This figure reflects the hypothesized relationships between ITC usage patterns, on the one hand, and MSDs and visual discomfort, on the other. It is postulated that usage patterns influence the emergence of these disorders, with groups that use the devices more showing more incidence of disorders. Training measures in preventive and instrumental measures in the use of devices (adequate devices, ergonomically adequate furniture, and ergonomic auxiliary elements) are expected to be related to lower levels of incidence of the disorders. Usage patterns modulate the buffer effect and effectiveness of the measures. The ratio of workers’ pauses per hour, their average duration, and their occurrence directly reduce MSDs and visual disorders, and they are conceptualized analytically as covariates. Visual discomfort and MSDs may also be related to age, and MSDs to the performance of intense physical exercise outside the work setting. These variables could thus distort the observed relationships, as they are not necessarily balanced in the different groups of ICT usage. Therefore, they have also been included as covariates, although, unlike the ratio of pauses per hour and their duration, they are mainly considered as extraneous variables to be controlled, not as conceptually relevant variables.

## 3. Materials and Methods

### 3.1. Subjects

A non-probabilistic sample, made up of 1259 workers from different productive sectors of Spanish organizations was performed. For sample selection, the research team contacted the health and safety managers from a total of 10,011 companies by email. The companies were in a database provided previously by the team members, which included the organizations with which the members had maintained training and consultancy activities in recent years. The email message presented the objectives of the study and requested their collaboration. A total of 512 prevention managers of the 10,011 contacted companies agreed to collaborate and sent the designed instrument to the workers of their companies, requesting their collaboration. 1259 workers responded following this request.

The sample comprises 708 women (56.2%) and 551 men (43.8%). The mean age was 43.60 years, SD = 8.72, with a range of 19 to 67 years. The workers are distributed in the following activity sectors: services (*n* = 875; 69.5%), industry (*n* = 225; 17.9%), construction (*n* = 124; 9.8%), and agriculture (*n* = 35; 2.8%). The types of jobs performed were: management, administration, reception (*n* = 778; 62.4%), services and activities (*n* = 61; 4.8%), production, operator (*n* = 56; 4.4%), and commercial department (*n* = 26; 2.1%).

### 3.2. Variables and Instruments

A structured online questionnaire was used, with a total of 64 items with closed and open questions. The target variables and their indicators are listed in Table 1.

### 3.3. Procedure

The health and safety managers who agreed to participate in the study received a link to the designed questionnaire. The prevention services managers sent an email to the workers, presenting the main objectives of the study, requesting their collaboration, and ensuring the anonymity of their responses. The questionnaire was completed anonymously and did not include any identification or personal or company data. The workers completed the questionnaire during the months of October and November of 2017. The responses were collected directly in the database linked to the questionnaire. Study procedures were consensuated with those in charge of the Spanish National Institute of Safety and Health at Work and obtained previous ethical approval by the Committee of the involved academic institution. 

### 3.4. Data Analysis

The data analysis was performed as follows: 1. Prior descriptive analysis, including calculation of absolute and relative frequencies for categorical variables and centrality and deviation measures for numerical variables; analysis of the psychometric properties of the visual discomfort scale. 2. Exploratory cluster analysis with k-means algorithm to define the different forms of combined usage of ICTs in the sample, considering both the type of device and its usage time. 3. Development of an initial Multivariate Analysis of Variance (MANOVA) model that included all the relationships and indicators contemplated in the theoretical model; estimation of the strength of relationships between single and interaction terms, covariates, and the dependent variables 4. Elaboration of a refined MANOVA model in which covariates and factors that yielded no significant relationships with the dependent variables were eliminated. The analysis was carried out with the IBM^®^-SPSS Statistics^®^ package (IBM Corporation, Armonk, NY, USA).

## 4. Results

### 4.1. Descriptive Statistics and Reliability Results

Table 2, Table 3 and Table 4 present the descriptive results of the target variables of the study. In the case of the visual discomfort scale, a reliability indicator is provided.

In relation to the place where ICTs devices were used for work-related tasks, 53.2 % of the sample (*n* = 672) used ICT devices only at a fixed workplace. Other options (non-exclusive) included “Home office” (14.0%; *n* = 177); “Other places at home” (4.1%; *n* = 52); “Public spaces” (10.7%, *n* = 135) and “Other spaces” (14.6%; *n* = 184). The performance of physical activities outside of working hours obtained a mean of 3.39/10 and a standard deviation of 2.89/10.

### 4.2. Cluster Analysis of the Usage Patterns of ICTs

The items of the questionnaire directly describe the daily usage time of each one of the different devices. However, these indicators are not valid to use to explore the relationships of ICT usage separately through occupational health indicators. This is because each worker can combine the use of diverse devices differently, and this affects workers’ occupational health. Therefore, taking as reference the analytical strategies based on exposure combinations [14] we empirically determined the different forms of combined usage of ICTs that occurred in the sample, considering both the type of device and its usage time. For this purpose, we used cluster analysis through the k-means algorithm, including the average usage time of each device, desktop computer (DC), laptop computer (LC), smartphone (SPh) and tablet (Tb) as classification variables. In view of the empirical results, a 5-group solution was chosen, which represents a good balance between group homogeneity and intergroup diversity. The description of each usage pattern and the label assigned are presented in Table 5. 

The detected groups have the following characteristics and denominations

- Low ICT Usage: Group of workers who do not use ICTs very much, with one third of workers in a fixed post.
- Preferred use of DC: group of workers that preferentially uses the DC and works in a predominantly fixed post.
- Combined use of DC and SPh: group of workers who use the DC and SPh equally with almost one half of the workers in a fixed post.
- Combined use of LC and SPh: Group of workers that uses a LC and a SPh equally, with a large majority of the workers in a mobile post.
- Combined use of DC and LC: Group of workers who use a DC and a LC equally, with one third of the workers in a fixed post.

The socio-demographic characteristics and job profile of each ICTs usage group is exposed in Table A1, which is included in the Appendix A. The different usage groups do not show contrastable differences regarding, sex, sector of activity, and job tenure. In contrast, the variable age showed a global relationship with ICTs usage groups (F = 1.95; *p* = 0.035) and, specifically, was slightly higher for Low ICTs usage group. The type of job varied also across ICTs usage patterns (LR = 155.0; *p* < 0.001). In this sense, preferred use of DC was higher than expected among management, administration and reception jobs, combined use of LC and SPh was higher in services and activities and combined use of DC and SPh yielded higher frequency among jobs linked to commercial department.

### 4.3. Relationships of Usage Patterns with MSDs and Visual Discomfort

To explore the relationships between usage patterns of ICT, MSDs, and visual discomfort, we used a MANOVA model that globally presents all the relationships contemplated in the theoretical model shown in Figure 1. The terms included in this initial model are shown in the Appendix A in Table A2. Based on this global model, we constructed a refined model in which: (1) the covariates that did not affect the relationships between the factors and the dependent variables empirically were eliminated, taking as elimination criteria the absence of significant relationship between the covariable and the dependent variables (*p* < 0.05) and the absence of change in the significance levels (from non-significant to significant or vice-versa) between any dependent variable and any factor when the covariate was removed from the model; (2) the factors (simple and of interaction) that did not reach significant relationships (*p* < 0.05) with the dependent variables were eliminated. 

The variables finally included in the refined MANOVA model and the results referring to the multivariate tests of this model are shown in Table 6. They indicate the extent to which each of the factors and covariates are related to the set of dependent target variables. 

**Figure 2:**
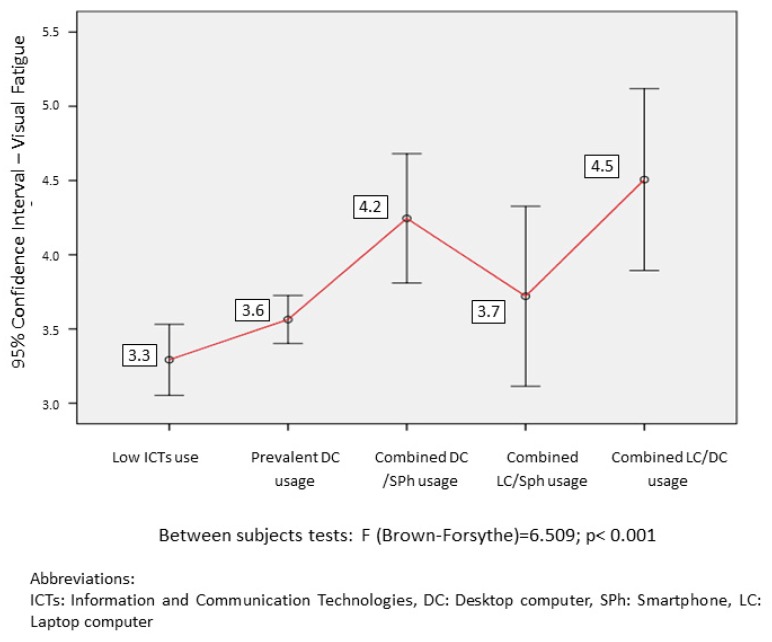
Relationships between visual discomfort and ICT usage pattern.

**Figure 3:**
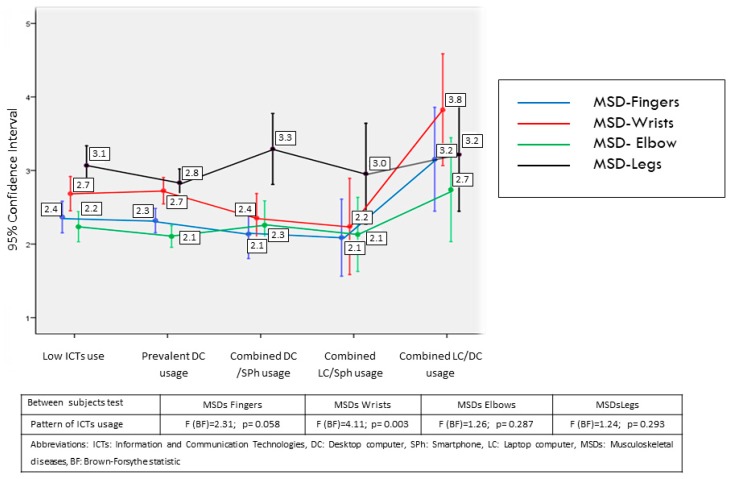
Relationships between MSDs and ICT usage pattern (1).

**Figure 4:**
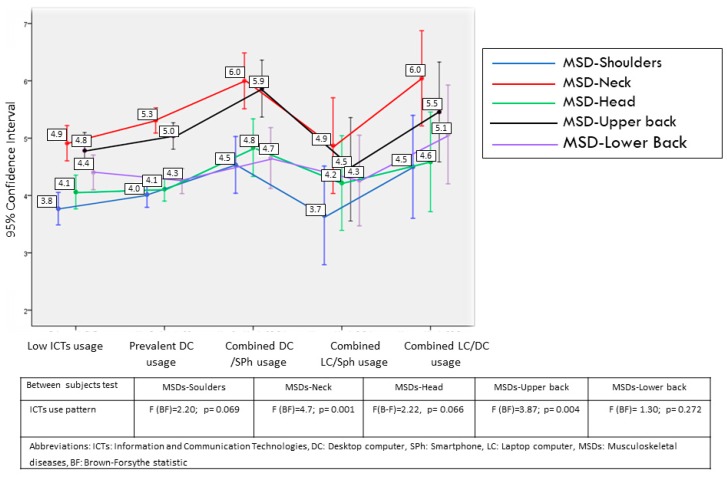
Relationships between MSDs and ICT usage pattern (2).

The results obtained are detailed more specifically in Figure 2, Figure 3 and Figure 4 and Table 7 and Table 8, which, in addition to the means of each group, provide the mean contrast statistics. To avoid the distortions linked to the non-homogeneity of variance between groups, the Brown-Forsythe statistic, a robust estimator of the difference of means unaffected by heteroscedasticity [15], was used. The figures show the relationship between ICT usage pattern, visual discomfort, and MSDs. The tables present the relation of these dependent variables with the other factors.

With regard to covariates with preventive implications, the ratio of pauses per hour had negative correlations with visual discomfort (*r =* −0.088, *p* = 0.002) and various indicators of MSDs: shoulders (*r =* −0.070, *p* = 0.014), neck (*r =* −0.092, *p* = 0.001), head (*r =* −0.100, *p* < 0.001), and upper back (*r =* −0.086, *p* = 0.003). This shows that a higher frequency of pauses is related to a lower incidence of visual discomfort.

Regarding the relationship between ICT usage pattern and the level of visual discomfort (Figure 2), there is more visual discomfort in the groups with combined usage of DC-SPh and DC-LC compared to the groups with low ICT usage and exclusive usage of DC. The total usage time of devices has a relevant correlation with visual discomfort (*r* = 0.266, *p* < 0.001). When examining the usage time of each device separately, the device with the highest ratio of visual discomfort is the DC (*r* = 0.146, *p* < 0.001), followed by the Sph (*r* = 0.120, *p* < 0.001), Tb (*r* = 0.089, *p* = 0.002) and LC (*r* = 0.066, *p* = 0.018). 

With regard to the relation of usage patterns with MSDs (Figure 3 and Figure 4), differences are found in the following indicators: increased incidence of wrist discomfort in workers with combined use of DC-LC compared to the other groups, and more incidence of neck and upper back problems in groups with combined usage of DC-SPh and DC-LC compared with the remaining groups. The correlation between global time dedicated to ICTs and incidence of MSDs is verifiable although it is moderate for neck (*r* = 0.187, *p* < 0.001) and upper back problems (*r* = 0.151, *p* < 0.001). Analyzed separately, desktop computer usage time has the highest correlations with these problems compared with the usage of other devices. Thus, the usage of DC has correlations of *r* = 0.159 (*p* < 0.001) and *r* = 0.126 (*p* < 0.001) with neck and upper back pain, respectively. The lower relationship of laptop usage with both disorders is notable (*r* = 0.027, *p* = 0.337 and *r* = 0.015, *p* = 0.586); tablet (*r* = 0.069, *p* = 0.015 and *r* = 0.062, *p* = 0.027); and smartphone (*r* = 0.080, *p* = 0.005 and *r* = 0.071, *p* = 0.012).

With regard to preventive measures (Table 7 and Table 8), the existence of task-appropriate devices is related to lower levels of visual discomfort and lower incidence of MSDs in all body areas considered. The use of elements to improve posture in front of the computer, however, only has verifiable relations with upper back pain, and in the opposite direction than that expected, a fact we will analyze in the discussion. The training measures are related to a lower incidence of numerous MSDs indicators, and this relationship is clearer for general training measures than for those referring to specific portable devices. In any case, the effect of training measures has a different relation with the reduction of MSDs as a function of the ICT usage pattern. In four of the usage pattern groups (low ICT usage, exclusive DC usage, combined DC-Sph usage, and combined LC-Sph usage), the existence of training measures is related to a lower incidence of disorders. However, in the group of combined DC-LC usage, the training measures are related to a higher incidence of diverse disorders, a fact that we will analyze in the discussion. Although the overall differences in the training received in the different ICT usage groups are found only at the limit of significance (χ^2^ = 9.23, *p* = 0.056), the DC-LC usage group stands out for having had less training opportunities (71.7% compared to 84.2% for all the groups; Adjusted Standardized Residual = −2.4, *p* < 0.05).

## 5. Discussion

In the present study, a new approach to the study of the ICT usage at work was used, drawing on the need to operationalize the combined usage of different devices. Our results show that it is possible to identify different patterns of combined ICT usage in the work setting, which imply varying degrees of mobility and total time dedicated to ICT usage. Our results also show that different usage patterns lead to differences in the levels of incidence of visual discomfort and MSDs. Lastly, they show the relation between different technical and training measures and the incidence of the aforementioned disorders. 

In relation to ICT usage patterns in the work setting, we have identified five different usage forms. The concordance of this classification with some of the ICT usage patterns in the working population detected in other studies [2,16,17] and its relation with the incidence of health indicators obtained in our study support its relevance. The group of workers with a comparatively low ICT exceeds, in any case, four hours of usage, indicating the extent of ICT in many work settings. This group is characterized by a higher level of mobility than other groups, and its almost exclusive use of portable devices. This indicates that the mobility of certain workers is not necessarily related to the use of ICT devices. The second group includes workers who use the desktop computer exclusively, with clearly fixed posts and with an ICT usage time equal to a regular workday. The three remaining groups of ICT usage pattern reflect the emerging ICT forms that we mentioned in the introduction, as these workers combine the different devices to varying degrees. It is noteworthy that these three groups accumulate a high amount of ICT usage time in all cases. This high number of hours shows that work activities extend beyond the formal workday established by the common rules in western contexts. Of these three groups, one of them is consistent with the pattern of the so-called multi-locational eWorkers [16], as their use of ICTs corresponds almost exclusively to mobile devices (LC, Sph, Tb) and most of them work at mobile posts. The group of combined use of LC and DC is of special interest in preventive terms, as it is especially exposed to the analyzed disorders, as we comment on below. 

ICT usage patterns showed isolated relationships with socio-demographic characteristics, (specifically age) and job characteristics (specifically type of job). The fact that a non-probabilistic sample was used does not allow making direct inferences about socio-demographic and job profiles of ICT patterns of usage in the general population of workers. However, evidence of age differences among ICTs usage groups in our sample made it necessary to control this variable when performing intergroup comparisons of VF and MSDs. 

The results show the usefulness of controlling for the variables age and physical exercise outside of the work setting in studies of the impact of the usage of ICT devices on visual health and MSDs, as these variables have contrasting relations, albeit low, with such usage. In other works, the relationship of age with visual discomfort in ICT users has been shown [18]. There is also evidence of the link between age and MSDs, especially in the cervical area, in workers who use ICTs in different sectors [6,19,20,21,22]. The inverse relationship found between the ratio of pauses per hour and visual discomfort provides evidence of the effect of pauses for the prevention of visual disorders in workers who use ICTs, although the evidence is still considered insufficient in the literature [23,24]. Similarly, the inverse relationship between the pause-taking ratio and the incidence of MSDs supports the preventive relationship of pauses with MSDs, which is considered inconsistent or incomplete according to the reviews by Bongers et al. [25], Brewer et al. [23] and Stock et al. [26]. The absence of a relationship between the average duration of pauses, their number, and their multiplication with the disorders could be due to the generic nature of the indicators of pauses used. A more detailed record of pauses in controlled conditions is probably required to adequately show their relationship with the different ailments.

The results show that there are clear differences in the level of visual discomfort between the different groups of ICT usage, with greater incidence of visual discomfort in the groups of combined DC-SPh and DC-LC usage. This effect is detected despite subtracting the influence of the ratio of pauses per hour made in the analyses. A substantive part of this relationship could be explained by the differences in the daily time devoted to the usage of the devices for each of the groups, which reaches similar and very high levels in the groups of DC-Sph, LC-Sph, and DC-LC. The relationship observed between total device usage time and visual discomfort supports the evidence already shown in various studies [24,27]. The fact that despite its extended ICT usage time, the group that combines P-Sph shows less incidence of visual discomfort could indicate that the combined usage of different devices implies less sustained usage times of video display terminals, thus protecting users from visual discomfort. Although the group of combined DC-LC usage shows greater incidence of visual discomfort, the error range of the mean score in this group does not allow establishing generalizable inferences about their differences with the remaining groups. 

The highest incidence found of MSDs in the trunk, head, and shoulders with regard to the limbs (fingers, wrists, elbows, and legs) and, especially the high incidence of MSDs in the neck and upper back, coincide with recent reports that back and cervical pains, mainly caused by occupational ergonomic hazards, have become the main cause of disability in Spain in 2016 [28]. Xie et al. [13] also indicate from the studies carried out in different contexts that MSDs in the neck have the highest prevalence in users of handled devices compared to other body areas. All this points to the need to prioritize preventive measures aimed at reducing the emergence of MSDs in this body area.

The different incidence of MSDs found among the different ICT usage groups in some specific body areas also has some interesting implications. Firstly, the increased incidence of wrist problems in workers with combined usage of DC and LC compared to the other groups, including those with extensive use of mobile devices, is notable, although the degree of impairment is moderate. In the light of the existing works, the incidence of MSDs in wrists is linked to the use of the mouse in the devices, and there is no evidence that the use of the keyboard is related to these problems [24,29,30]. This would explain the lower incidence of MSDs in usage patterns that, despite a high amount of ICT usage time, include a relevant amount of devices. This lack of wrist problems in workers who use handled devices is also reported in the recent review of Xie et al. [13].

Secondly, the contrasting differences in the appearance of MSDs between the different ICT usage patterns in two body areas are noteworthy: upper part of the head and neck. Thus, there is a trend towards a higher incidence of neck and upper-back problems in groups of combined usage of DC-Sph and DC-LC compared to the remaining groups. The combined P-Sph group, despite the high usage time, yields similar levels to groups with less ICT usage. In general, the relationship between high usage of video display terminals, on the one hand, and cervical and upper back pain, on the other, is well established in the literature [18,22,24,30]. In the specific case of the handled devices, there are various works showing this, although it is still considered inconsistent [5,13]. As shown, the relationship between global time dedicated to ICTs and incidence of MSDs was contrastable, although moderate for neck and upper back pain. In any case, once again, the usage time of the DC had a stronger relationship with these problems compared to the usage of other devices. These results are not consistent with the study of So et al. [5], one of the scarce works that have analyzed conjointly the relationship between different ICT devices and MSDs. This work found the greatest relationship between usage time and MSDs in the neck for the Sph. However, the recent study of Taib et al. [31], performed through electromyographic recordings, points out that, compared to DC and LC use, the lowest activity for all muscles was obtained during the use of a Tb or Sph. This last work supports the greater vulnerability of workers who combine DC and LC, which we have shown in our results. The usage pattern that includes only mobile devices, again, does not seem especially susceptible to this type of problems in relation to the remaining groups. This could show the protective effect of variability and alternation in the use of devices. 

It is noteworthy that, when examining the relationship between different technical measures and the incidence of visual discomfort and MSDs, when workers report that they have appropriate devices or equipment, the incidence of both these disorders is lower. This result is consistent with the existing literature, which points out that the ergonomic adequacy of ICT devices is related to a lower incidence of MSDs in different body areas, although the causal evidence of this relationship is still inconsistent [23,24,30,32].

The existing literature presents some evidence of the positive effect of ergonomic training in the modification of the habits of the workers who use ICTs [33,34] and, consequently, the reduction of visual discomfort and MSDs [35,36]. The results obtained show that having received general training in the prevention of occupational hazards in the workplace is related to a lower incidence of almost all the different disorders, supporting the evidence about the potential usefulness of such training. As the DC-LC usage group stands out for having received less training measures, this could help to explain, along with the other factors, this group’s greater incidence of disorders. In turn, the results show that specific training in the use of mobile devices is related to a lower incidence of different indicators. 

In any case, the results should be considered in the light of some limitations. Firstly, the use of a non-probabilistic sample allows exploring relations between usage patterns and physical disorders but it does not allow making inferences about the socio-demographic profile or the incidence in general population of these groups. Secondly, it should be taken into account that this is a first exploratory approach to the patterns of global ICT usage in the work setting, carried out through global indicators collected through self-report. This allows a first rough approach in a broad convenience sample to the general usage patterns and their relation with certain disorders, but not a specific analysis of the effect of postural elements or specific ergonomic devices. Although we drew on a defined conceptual approach, which hypothesizes the direction of the influence between the variables, the type of design used does not allow establishing causal relations between the selected indicators. Therefore, these findings should be supplemented by studies that more reliably analyze in necessarily smaller samples the relationship trends we found. The use of a broad sample in which the usage patterns were not predefined has also conditioned the analysis, as we obtained important differences in the size of the different groups. We tried to compensate for these differences by using robust indicators, thereby adopting a conservative approach to the detection of the relationships. This implies that certain differences may have been masked, especially those referring to smaller groups. In addition, we note that this study analyzed the effects on physical health of new usage patterns of ICTs based on a set of variables related to times and forms of usage, preventive measures, and a reduced set of individual variables. However, some additional variables that have shown a link in the literature with the expression of physical disorders have not yet been included. These include the micro-climate, [37,38], various organizational factors such as design, organizational policies, management practices and career opportunities [25,39,40,41] and certain individual factors, such as anxiety, mood disorders or psychophysical distress [39,40,41,42]. Likewise, the use of devices intended for leisure activities was not considered. In this sense, this study can serve as a starting point to complement the proposed conceptual framework with additional factors at the physical, organizational, and individual levels. This could increase the explanatory capacity of the onset and expression of visual and musculoskeletal disorders in workers using ICTs.

## 6. Conclusions

It is possible to detect in the work setting different ICT usage patterns that include combinations of fixed and mobile devices. Some of these patterns detected in the Spanish context reflect an extensive hourly investment, which exceeds the recommended maximum of daily 7/8 working hours. In the entire working population, MSDs in the neck, which show a considerably greater incidence than those of other body areas, stand out and should be the priority object of preventive action. 

The ICT usage pattern with the highest incidence of visual disorders and MSDs is the one that combines the use of laptop and desktop computers for extensive working hours. However, the group of workers primarily using mobile devices and working mostly in mobile posts does not appear to be particularly vulnerable to these disorders. Both visual discomfort and different MSDs are related to the usage time of the devices, although the combination of various devices, such as the case of mobile workers, seems less damaging than the prolonged use of a small number of devices. Therefore, the current ICT usage panorama in the working population requires paying attention to new forms of intensive device usage, which does not simply correspond to the usage of mobile devices and which can have significant impact on workers’ health.

It is necessary to control for the variables age and physical exercise outside the work setting in studies that examine the impact of ICT use on visual health and MSDs, as these variables have verifiable, albeit low, relations with visual discomfort and MSDs. Not taking them into account could alter the results of observational studies.

The ratio of workers’ pauses per hour in the usage of devices is related to a lower incidence of visual discomfort and MSDs in the shoulders, neck, head, and upper back, a fact that supports the relevance of implementing pauses as a potentially effective preventive action. Similarly, there is a lower incidence of visual discomfort and MSDs, especially wrist and neck problems, when workers report that they have adequate devices. Receiving general training on prevention of occupational hazards in the workplace is related to a lower incidence of almost all the different disorders, supporting the potential usefulness of such training. This relationship is especially strong in visual discomfort and MSDs that affect the neck and head.

The current panorama of ICT usage is thus shown to be complex, and must be addressed using specific and detailed empirical analysis of the different forms of combined use of mobile and fixed devices and their impact on workers’ health. This precise evaluation will allow for adequately directing the preventive actions in a working population that is clearly affected by some MSDs.

## Figures and Tables

**Table 1:** Variables and indicators.

Socio-demographic data:	Sex, age
Job data:	Sector of activity, type of job, job tenure.
ICT usage in the work activity:	Average daily usage time (hours) of the following types of technologies for work-related activities: desktop, laptop, tablet, and smartphone. Average number of daily pauses in ICT usage (informed by subjects) Average duration of pauses in ICT usage (informed by subjects) Place of usage of ICT devices (Only fixed posts at work; home office; other spaces at home, public places; other spaces)
Preventive measures taken	Technical measures (TM): Check-list (Yes/No) about the availability of: Furniture that allows adopting an adequate posture to perform the task (TM1). Appropriate devices or equipment for the task being performed (TM2). Lecterns, footrests or other elements that contribute to improve posture in front of the computer (TM3). Accessory element(s) (lecterns, supports for tablets, ...) that allow adopting an adequate posture when using smartphones and tablets (TM4).
	Training measures: (TRM): check-list (Yes/No) about the availability in the company of: Training/general information about Occupational Hazard Prevention (TRM1) Computer-related training (TRM2) Training related to the use of other devices (TRM3)
Indicators of occupational health	Visual discomfort: rating scale from 1 (lowest intensity) to 10 (highest intensity) of seven descriptives to collect a subjective rating of visual discomfort after device use (Laubli, Hunting, & Grandjean, 1981; Saito et al., 1994): (a) “My eyes feel tired (visual fatigue)”; (b) “Eyes dry, irritated or burning”; (c) “Eye pain”; (d) “Hard to focus”; (e) “Double vision on screen”; (f) “Flicker vision”; and (g) “Headache”.
	Musculo skeletal disorders (MSDs): Nordic Musculoskeletal Questionnaire (NMQ), (Baron, Hales & Hurrell, 1996). Check-list of 9 items referring to pain or discomfort perceived by workers in different body parts suffered for seven days following the use of ICTs. Response scale ranging from 1 (no pain or discomfort) to 10 (maximum intensity of pain or discomfort).
Performance of physical activity out of the work setting:	Item that shows the extent to which workers practice hobbies or intensive sports activities, play musical instruments or practice activities with instruments that produce vibrations (lawnmower, motor saw, DIY tools, etc.) in their free time. Scale from 0 to 10 points.

**Table 2:** Descriptive results (1): Centrality and deviation indicators of ICT Usage.

ICT Usage	Mean	Standard Deviation
Average time (hours) of daily usage of desktop computer	4.44	2.99
Average time (hours) of daily usage of laptop	1.15	2.14
Average time (hours) of daily usage of Tablet	0.21	0.83
Average time (hours) of daily usage of Smartphone	1.79	2.47
Pause ratio for total hours of ICT usage (pauses/hour)	0.73	1.05
Average time per pause (minutes) in ICT usage	3.87	17.39

**Table 3:** Descriptive results (2): absolute and relative frequencies of the preventive measures taken.

Preventive Measures	No		Yes	
	*n*	%	*n*	%
Technical Measures (TM)				
TM1. Appropriate devices or equipment for the task being performed	490	(39.0%)	765	(61.0%)
TM2. The company carried out a specific occupational hazard assessment for working with these devices	535	(42.6%)	720	(57.4%)
TM3. Furniture that allows adopting an adequate posture to perform the task	412	(32.8%)	843	(67.2%)
TM4. Lecterns, footrests or other contributing elements to improve posture in front of the computer	743	(60.0%)	496	(40.0%)
TM5. accessory element/s (lecterns, stands...) that allow adopting an adequate posture when using smartphones/tablets	1024	(81.6%)	231	(18.4%)
Training measures (TRM)				
TRM1. General training on prevention of occupational hazards	199	(15.8%)	1060	(84.2%)
TRM2. Training related to computer use	748	(59.4%)	511	(40.6%)
TRM3. Training related to the use of other devices	1067	(84.7%)	192	(15.3%)

**Table 4:** Descriptive results (3): Centrality, deviation of Visual discomfort and MSDs measures, as well as reliability indicators of Visual discomfort scale.

Visual Discomfort and MSDs Measures		Mean	Standard Deviation	Reliability (Cronbach’s Alpha)
Visual discomfort: From 1 (lowest intensity) to 10 (highest intensity)		3.60	2.25	0.905
MSDs: From 1 (no pain or discomfort) to 10 (maximum intensity of pain or discomfort)	MSDs Fingers	2.34	2.12	-
	MSDs Wrists	2.70	2.30	-
	MSDs Elbows	2.18	1.97	-
	MSDs Shoulders	4.00	2.89	-
	MSDs Neck	5.28	2.92	-
	MSDs Head	4.19	2.82	-
	MSDs Upper Back	5.05	3.04	-
	MSDs Lower Back	4.37	2.91	-
	MSDs Legs	2.97	2.53	-

Abbreviations: MSDs: Musculoskeletal Disorders.

**Table 5:** ICT Usage Pattern. Cluster analysis results.

		ICT Usage Pattern				
Usage of Specific Devices and Mobility		Low ICT Usage	Preferential DC Usage	Combined DC-Sph Usage	Combined LC-Sph Usage	Combined DC-LP Usage
Total Frequency	Row% (*n*)	29.0 (365)	53.1 (669)	10.6 (133)	3.7 (46)	3.7 (46)
		Mean	Mean	Mean	Mean	Mean
Average usage time (hours) of different devices at work	Desk Computer	0.7	6.3	6.6	.5	5.2
	Laptop Computer	2.2	0.2	0.6	5.3	5.0
	Tablet	0.2	0.1	0.3	1.6	0.8
	Smart Phone	1.3	0.6	6.7	7.7	2.6
		% (*n*)	% (*n*)	% (*n*)	% (*n*)	% (*n*)
Mobility in the work post	100% in a fixed physical post	32.9 (120)	69.5 (465)	47.4 (63)	19.6 (9)	32.6 (15)
	75% fixed-25% mobile	20.8 (76)	19.7 (132)	21.8 (29)	19.6 (9)	28.3 (13)
	50% fixed-50% mobile	26.3 (96)	9.9 (66)	21.8 (29)	37.0 (17)	32.6 (15)
	25% fixed-75% mobile	12.9 (47)	0.9 (6)	4.5 (6)	15.2 (7)	6.5 (3)
	100% Mobile	7.1 (26)	0.0 (0)	4.5 (6)	8.7 (4)	0.0 (0)

Abbreviations: ICTs: Information and Communication Technologies. SPh: Smartphone. DC: Desktop Computer. LC: Laptop Computer. Tb: Tablet.

**Table 6:** Relationship between visual discomfort, MSDs, ICT usage patterns, and preventive measures. MANOVA: Multivariate Tests.

Dependent Variables	Factors/Covariates with Relevant Relationships	Multivariate Tests (Global Relationship with Dependent Variables)
Visual Discomfort MSDs	FACTORS	
	ICT Usage Pattern	F = 37.78 (*p* < 0.001)
	TM1. Adequate ICT devices adapted to task requirements	F = 5.56 (*p* < 0.001)
	TM4. Accessory elements that improve posture	F = 3.63 (*p* < 0.001)
	TRM1. General hazard prevention training	F = 2.65 (*p* = 0.003)
	TRM3. Specific ICT hazard prevention training	F = 2.09 (*p* = 0.031)
	TRM3. Specific ICT hazard prevention training	F = 1.43 (*p* = 0.039)
	COVARIATES	
	Age	F = 6.46 (*p* < 0.001)
	Ratio of pauses per hour	F = 2.05 (*p* = 0.026)
	Intense exercise out of work	F = 2.40 (*p* = 0.008)

Abbreviations: MSDs: Musculoskeletal diseases. TM: Technical measure. TRM: Training measure.

**Table 7:** Relations between preventive measures, visual discomfort, and MSDs (1).

Preventive Measures		Visual Discomfort	MSDs Fingers	MSDs Wrists	MSDs Elbows	MSDs Legs
		Mean	Mean	Mean	Mean	Mean
TM1. Appropriate devices or equipment for the task being performed	No	3.96	2.70	3.18	2.38	3.39
	Yes	3.36	2.09	2.38	2.05	2.69
	F (B-F)	20.8	23.26	33.79	8.42	21.97
		*p* < 0.001	*p* < 0.001	*p* < 0.001	*p* = 0.004	*p* < 0.001
TM4. Lecterns, footrests, or other elements contributing to improve posture in front of the computer	No	3.50	2.39	2.63	2.20	2.92
	Yes	3.76	2.26	2.82	2.17	3.04
	F (B-F)	3.78	1.08	2.20	0.045	0.651
		*p* = 0.052	*p* = 0.297	*p* = 0.138	*p* = 0.831	*p* = 0.420
TRM1. General Training	No	4.20	2.68	3.03	2.56	3.12
	Yes	3.48	2.27	2.63	2.11	2.94
	F(B-F)	17.77	5.31	4.22	7.22	0.84
		*p* < 0.001	*p* = 0.022	*p* = 0.041	*p* = 0.008	*p* = 0.362
TRM3. Training related to the use of other devices	No	3.65	2.37	2.74	2.19	3.04
	Yes	3.32	2.15	2.44	2.16	2.55
	F(B-F)	3.82	2.08	2.96	0.033	7.31
		*p* = 0.052	*p* = 0.150	*p* = 0.086	*p* = 0.855	*p* = 0.007

Abbreviations: TM: Technical measure. TRM: Training measure. MSDs: Musculoskeletal disorder. F(BF): Value of the Brown-Forsythe F statistic.

**Table 8:** Relations between preventive measures. Visual discomfort. And musculoskeletal disorders (2).

Preventive Measures		MSDs Shoulders	MSDs Neck	MSDs Head	MSDs Upper Back	MSDs Lower Back
		Mean	Mean	Mean	Mean	Mean
P 8.1.1 Devices or equipment appropriate for the task being performed	No	4.53	5.86	4.69	5.48	4.91
	Yes	3.67	4.91	3.88	4.77	4.01
	F(B-F)	26.26	32.14	24.63	16.12	28.55
		*p* < 0.001	*p* < 0.001	*p* < 0.001	*p* < 0.001	*p* < 0.001
P 8.1.4 Lecterns. footrests or other elements contributing to improve posture in front of the computer	No	3.92	5.17	4.20	4.88	4.42
	Yes	4.20	5.51	4.22	5.31	4.30
	F(B-F)	2.76	3.79	0.012	5.90	0.466
		*p* = 0.097	*p* = 0.052	*p* = 0.914	*p* = 0.015	*p* = 0.420
P8.3.1 General Training	No	4.52	6.04	4.95	5.49	4.91
	Yes	3.91	5.14	4.05	4.96	4.27
	F(B-F)	7.09	15.93	16.28	4.90	8.31
		*p* = 0.008	*p* < 0.001	*p* < 0.001	*p* = 0.028	*p* = 0.004
P 8.3.3 Training related to the use of other devices	No	4.06	5.35	4.30	5.14	4.47
	Yes	3.71	4.90	3.60	4.51	3.80
	F(B-F)	2.46	4.00	11.09	7.30	9.10
		*p* = 0.118	*p* = 0.046	*p* = 0.001	*p* = 0.007	*p* = 0.003

Abbreviations: TM: Technical measure. TRM: Training measure. MSDs: Musculoskeletal disorder. F(BF): Value of the Brown-Forsythe F statistic.

## Appendix A

**Table A1:** Socio-demographic characteristics and job profile of ICTs usage patterns.

		ICT Usage Pattern				
Socio-Demographic Characteristics and Job Profile		Low ICT Usage	Preferred Use of DC	Combined Use of DC and SPh	Combined Use of LC and SPh	Combined Use of DC and LC
		% (*n*)	% (*n*)	% (*n*)	% (*n*)	% (*n*)
Sex (LR = 6.47; *p* = 0.165)	Male	47.7 (174)	42.3 (283)	36.8(49)	47.8 (22)	50.0 (23)
	Female	52.3 (191)	57.7 (386)	63.2 (84)	52.2 (24)	50.0 (23)
Type of job (LR = 155.0; *p* < 0.001)	Management, administration, reception	43.3 (158)	75.9 (508) +	62.4 (83)	37.0 (17) −	47.8 (22) −
	Production, operator	9.6 (35)	1.5 (10) −	3.8 (5)	6.5 (3)	6.5 (3)
	Commercial department	1.6 (6)	1.5 (10)	4.5 (6) +	4.3 (2)	4.3 (2)
	Services and activities	8.2 (30)	2.5 (17) −	2.3 (3)	15.2 (7) +	8.7 (4)
	Others	37.3 (136)	18.5 (124) −	27.1 (36)	37.0 (17)	32.6 (15)
Sector of activity (LR = 15.3; *p* = 0.146)	Agriculture	3.3 (12)	2.8(19)	2.3 (3)	2.2 (1)	0.0 (0)
	Construction	12.3 (45)	9.3(62)	6.0 (8)	13.0 (6)	6.5 (3)
	Industry	15.1 (55)	20.2(135)	18.8 (25)	10.9 (5)	10.9 (5)
	Services	69.3 (253)	67.7(453)	72.9 (97)	73.9 (34)	82.6 (38) +
Job tenure (LR = 23.5; *p* = 0.100)	Less than 1 year	4.9 (18)	3.4(23)	3.0 (4)	2.2 (1)	8.7 (4)
	1−5 years	27.1 (99)	29.6(198)	34.6 (46)	45.7 (21) +	21.7 (10)
	6−10 years	12.3 (45)	13.5(90)	15.0 (20)	10.9 (5)	15.2 (7)
	11−15 years	18.4 (67)	15.2(102)	15.0 (20)	26.1 (12)	15.2 (7)
	>15 years	37.3 (136)	38.3 (256)	32.3 (43)	15.2 (7) −	39.1 (18)
Age (F = 1.95; *p* = 0.035)		Mean (SD)	Mean (SD)	SD	Mean (SD)	Mean (SD)
		44.68 (8.6)	42.89 (9.28)	43.56 (8.68)	41.50 (9.00)	41.87 (8.65)

Abbreviations: LR: Chi-square Likelihood Ratio. F: Snedecor’s F. +: Adjusted Standarized Residual >2. −: Adjusted Standarized Residual <2.

**Table A2:** Relationship between visual discomfort, MSDs, ICT usage patterns, and preventive measures. Initial model for MANOVA.

Dependent Variables	Factors	Covariates
Visual discomfort MSDs	ICT usage pattern TM1. Appropriate devices or equipment for the task being performed TM2. The company carried out a specific occupational hazard assessment for working with these devices TM3. Furniture that allows adopting an adequate posture to perform the task TM4. Lecterns, footrests, or other contributing elements to improve posture in front of the computer TM5. Accessory Element(s) (Lecterns, stands ...) in order to adopt an appropriate position when using smartphones/tablets TRM1. General training on prevention of occupational hazards TRM2. Training related to computer use TRM3. Training related to the use of other devices ICT usage pattern TM1 ICT usage pattern TM2 ICT usage pattern TM3 ICT usage pattern TM4 ICT usage pattern TM5 ICT usage pattern TRM1 ICT usage pattern TRM2 ICT usage pattern TRM3	Age Physical activity outside the work setting Average pause time Ratio pauses per hour Average pause time ratio pauses per hour

Abbreviations: MSDs: Musculoskeletal Diseases. TM: Technical measure. TRM: Training measure.
